# A new large-volume metal reference standard for radioactive waste management

**DOI:** 10.1093/rpd/ncv309

**Published:** 2015-05-13

**Authors:** F. Tzika, M. Hult, H. Stroh, G. Marissens, D. Arnold, O. Burda, P. Kovář, J. Suran, A. Listkowska, Z. Tyminski

**Affiliations:** 1 EC-JRC-IRMM, European Commission, Joint Research Centre, Institute for Reference Materials and Measurements, Retieseweg 111, Geel 2440, Belgium; 2 PTB, Physikalisch-Technische Bundesanstalt, Bundesallee 100, Braunschweig 38116, Germany; 3 CMI, Czech Metrology Institute, Radiová 1a, Praha 10 102 00, Czech Republic; 4 NCBJ, Narodowe Centrum Badań Jądrowych RC POLATOM, ul. Andrzeja Sołtana 7, Otwock-Świerk 05-400, Poland; 5 Present address: EC-JRC-ITU, Institute for Transuranium Elements, PO Box 2340, Karlsruhe 76125, Germany

## Abstract

A new large-volume metal reference standard has been developed. The intended use is for calibration of free-release radioactivity measurement systems and is made up of cast iron tubes placed inside a box of the size of a Euro-pallet (80 × 120 cm). The tubes contain certified activity concentrations of ^60^Co (0.290±0.006 Bq g^−1^) and ^110m^Ag (3.05±0.09 Bq g^−1^) (reference date: 30 September 2013). They were produced using centrifugal casting from a smelt into which ^60^Co was first added and then one piece of neutron irradiated silver wire was progressively diluted. The iron castings were machined to the desirable dimensions. The final material consists of 12 iron tubes of 20 cm outer diameter, 17.6 cm inner diameter, 40 cm length/height and 245.9 kg total mass. This paper describes the reference standard and the process of determining the reference activity values.

## INTRODUCTION

In the coming years, many old nuclear facilities will be decommissioned. This will generate large amounts of waste (mainly low-level waste). One obsolete reactor alone may generate 100 thousand tons of waste of metal and concrete. It is of great importance that radioactive waste be managed safely to avoid impact on workers, the general public and environment. It is also important to minimise radioactive waste and thereby reducing long-term costs by e.g. recycling as much as possible. One important step towards radioactive waste minimisation is to develop accurate and traceable radionuclide-specific measurement techniques for waste characterization and free release. To obtain reliable quantitative results from both waste characterisation and so-called free-release measurement facilities (FRMFs), it is essential that calibration standards of good quality are available.

In 2011, the joint research project ‘Metrology for Radioactive Waste Management’ or ‘MetroRWM’ [http://www.radwaste-emrp.eu/ (12 March 2015, date last accessed)] was launched in the framework of European Metrology Research Programme (EMRP) organised by Euramet. In this project, several European National Metrology Institutes worked together to develop methods for better measurements of radioactive waste. A prototype FRMF based on four HPGe-detectors was conceived and installed at the company ENVINET in the Czech Republic. In connection to this FRMF, three large-volume calibration standards for different types of waste were developed. This paper describes the development of one such calibration standard for metal waste including the two radionuclides ^110m^Ag and ^60^Co. These two nuclides were selected with the following reasoning: (a) they are both neutron activation products found in irradiated steel components of nuclear facilities, (b) they emit multiple gamma rays in the energy range of interest for efficiency calibration of clearance systems and (c) based on their physical and chemical properties, it was expected that their distributions in the iron matrix would be homogeneous with respect to the set requirements.

The calibration standard consists of 12 metal tubes placed in a special container of Euro-pallet size. The shape (tubes) of the standard matches geometries frequently met in practice, where often radioactive waste containers are filled with parts of contaminated/activated pipes from nuclear facilities, and further allows for dual use of the standard by e.g. filling cylindrical waste drums for calibration of waste characterisation systems. The criteria that governed the development of the metal calibration standard were the following:

- The material should be composed of homogeneous matrix and its apparent density (mass divided by occupying volume) should lie between 1 and 2 g cm^−3^.
- The total activity content of each radionuclide should be below its exempted activity value (10^5^ Bq for ^60^Co and 10^6^ Bq for ^110m^Ag)^(1)^ for easier handling of the standard.
- The ‘between-tubes’ homogeneity of the activity distribution should be better than 2 and 5 % for ^60^Co and ^110m^Ag, respectively.
- The ‘within-tube’ homogeneity of the activity distribution should be better than 2 and 5 % for ^60^Co and ^110m^Ag, respectively.
- The activity concentration (in Bq g^−1^) of each of the two radionuclides should be determined with an uncertainty of <10 % including inhomogeneity.

## MATERIALS

### Production of cast iron tubes and discs

**Figure 1.**
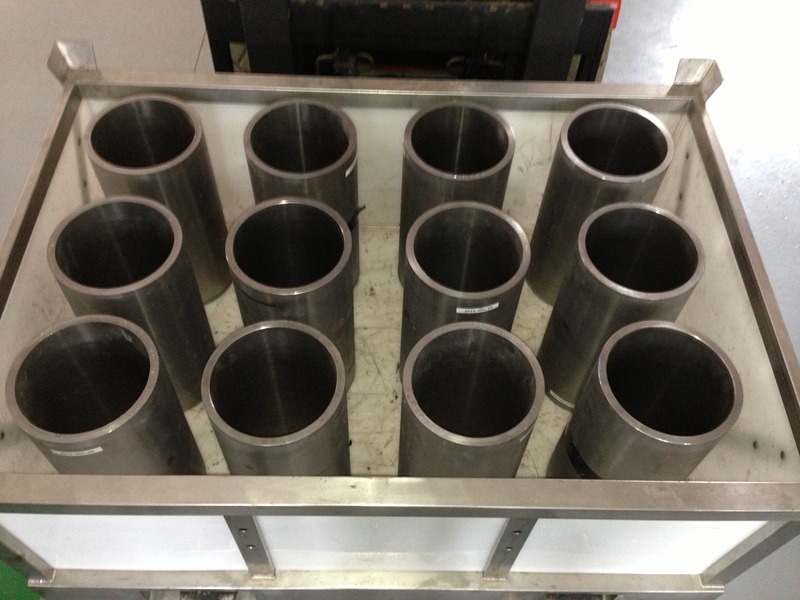
Photo of the reference standard with the 12 cast iron tubes inside the dedicated container.

The metal (grey cast iron with lamellar graphite, grade DIN1691 GG25) standard in this work was produced using horizontal centrifugal casting from a smelt in which a known activity of ^60^Co was added and one piece of neutron irradiated silver wire was progressively diluted. The minor elements present in a concentration of >0.1 % (by mass) are C (3.2 %), Si (1.7 %), P (0.2 %) and Mn (0.6 %). The material was produced by the Czech metallurgical company VUHZ that is specialised in casting iron. The metal standard has the geometrical configuration of tubes. An 80-cm-long tube was casted from each of the six basins/batches containing 95 kg of melted iron. The casting was in turn cut into two pieces that were machined to the desirable dimensions. The final material consisted of 12 iron tubes of 20 cm of outer diameter, 17.6 cm of inner diameter, 40 cm of length and 245.9 kg of total mass. The material was placed into a Euro-pallet-sized container (volume 120 × 80 × 40 cm^3^). Figure 1 shows the 12 tubes placed inside the dedicated container.

In addition, 1–2 discs were produced from the same smelt of each of the 6 castings to a total number of 10 discs. These discs were intended for accurate determination of the activity concentration in the material and for a test of the homogeneity of the activity distribution in the iron. The discs were not produced by centrifugal casting but simply by pouring the smelt in a disc-shaped mould. The discs were then machined to their final dimension of diameter of 80 mm, thickness of 10 mm and a mass of 362 g each.

Furthermore, from the outside of each tube, 3 samples (36 samples in total) of small metal chips (shavings) were sampled from the outer surface using a lathe. The three samples were taken from three heights, namely the top, middle and bottom, of each tube. During the machining process, the tube was in horizontal configuration and each sample was collected, from a machining length of 30 mm, using a paper under the lathe which the shavings fell on. In this manner, cross-contamination between different sampling heights was eliminated.

## MEASUREMENTS TO STUDY HOMOGENEITY

The homogeneity study of the activity distribution in the tubes material was based on measurements by gamma ray spectrometry of (a) the 10 discs cast from the same material as the tubes and (b) the 36 subsamples of shavings taken during machining of the individual tubes.

### Measurement of discs

All 10 discs were measured by JRC in the Radionuclide Metrology laboratories of JRC-IRMM. The discs were placed 20 mm above the end cap of a low-background HPGe-detector (‘Ge-T5’) with 40 % relative efficiency. In order to study the homogeneity within a disc, each disc was measured first in a ‘normal’ position and then upside down. Neither for ^60^Co nor for ^110m^Ag could any difference between the normal and the upside down measurement be distinguished.

**Figure 2.**
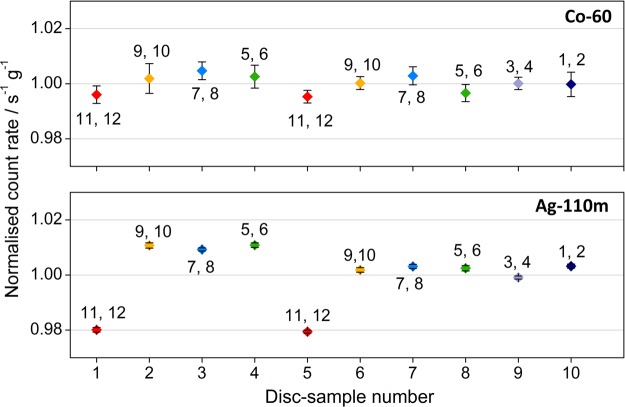
Normalized and decay-corrected count rate per gram of sample summed over the main peaks of ^60^Co and ^110m^Ag for the 10 discs that were cast from the same six smelts (indicated by different colours) as the tubes (indicated numbers). Error bars represent counting statistics, weighing, counting and decay time.

Figure 2 shows the sum of the count rate of the main peaks of ^110m^Ag: 658, 764, 885 and 938 keV, and ^60^Co: 1173 and 1332 keV, for each of the ten discs (taking for each disc the weighted mean of the normal and upside down measurements). The error bars in Figure 2 represent the combined standard uncertainty due to counting statistics, weighing, counting and decay times. The uncertainty introduced due to small differences in self-attenuation in slightly differing sample sizes is not shown. The latter was estimated by using Monte Carlo simulations to be <0.5 %. The relative standard deviations were 1.12 and 0.32 %, respectively, for ^110m^Ag and ^60^Co showing a more homogeneous distribution of ^60^Co in the six smelts as compared with the ^110m^Ag. The larger spread of ^110m^Ag data (Figure 2) is mainly influenced by the apparent lower concentration, by ∼3 %, of the nuclide measured in the discs (1 and 5) coming from the sixth basin as compared with the ones of other five basins. The relative difference in count rate between the highest and lowest values for the 10 discs was 3.1 and 0.9 %, respectively, for ^110m^Ag and ^60^Co.

Based on the disc measurements, the activity concentrations in the material for the two radionuclides in question meet the ‘between-tubes’ homogeneity criteria described in the introduction.

### Measurement of shavings

**Figure 3.**
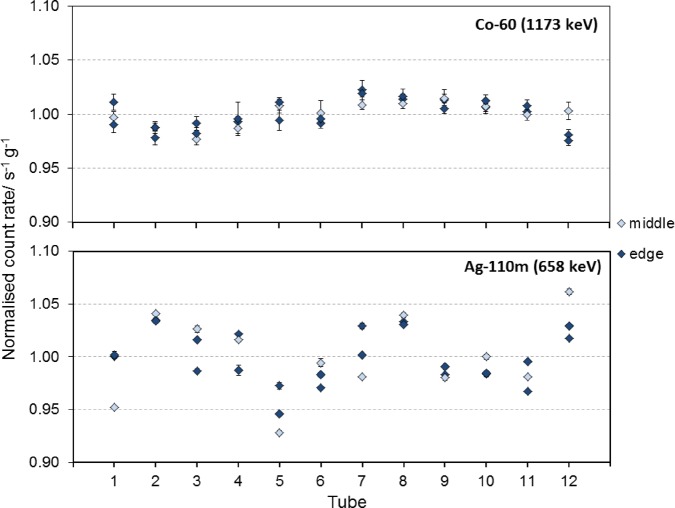
The decay-corrected count rate per gram of shaving samples normalized to the mean value of the count rate for ^60^Co and ^110m^Ag. Three samples, two from the edges and one from middle height, of each of 12 tubes are shown.

The 36 samples with shavings were measured by JRC-IRMM in the 225-m-deep underground laboratory HADES, where the muon-flux is reduced by a factor of 5000 compared with above ground^(2)^. An ultra-low-background HPGe-detector (‘Ge-3’) with a relative efficiency of 60 % was used for the purpose. The shavings were placed in a Teflon container with an inner diameter of 50 mm. The height of the shavings inside the container was typically 3.7 mm (minimum 3.6 and maximum 3.9 mm) and the mass typically 15.5 g. Figure 3 shows the count rate of the major line of ^110m^Ag at 658 keV and that of ^60^Co at 1173 keV for each of the 36 samples. The error bars in Figure 3 represent only the counting statistical uncertainty as the uncertainty of the mass and time is negligible in comparison. The only systematic uncertainties that can be of significance that are not accounted for are related to the sampling with the lathe and possible cross-contamination of shavings from other tubes. The relative uncertainty due to the sample height uncertainty (0.3 mm) was calculated using Monte Carlo simulations and corresponds to 1.5 % for ^110m^Ag and 1.0 % for ^60^Co.

The measurements of the samples of shavings confirmed the superior homogeneity of ^60^Co as compared with ^110m^Ag, which was observed during the measurements of discs. In the case of shavings, the relative standard deviations were 3.24 and 1.25 %, respectively, for ^110m^Ag and ^60^Co. The multiple subsamples (three replicates) per tube allowed for the evaluation of shavings results using analysis of variance (ANOVA) to derive a realistic inhomogeneity index. The calculated standard deviations between units, *s*_bb_, the ones within unit, *s*_wb_, and the uncertainties that could be hidden by the method repeatability, u_bb_*, are summarised in Table 1 for the two nuclides. The largest value of *s*_bb_ and u_bb_* was adopted as the uncertainty component introduced by inhomogeneity, u_hom_, in the reference activity concentration of each of the two nuclides in the tubes material (Table 1).

**Table 1.** ANOVA test results for ^60^Co and ^110m^Ag in shavings in comparison with standard deviations from all discs and shavings measurements (units %).

Radionuclide	^60^Co	^110m^Ag
Standard deviation of measurements of all 10 discs		
*s*_discs_	0.32	1.12
Standard deviation of measurements of all 36 shavings		
*s*_shavings_	1.25	3.24
ANOVA (shavings)		
*s*_bb_	1.06	2.39
*s*_wb_	0.76	1.83
u_bb_*	0.24	0.57
Adopted		
u_hom_	1.06	2.39

The results of the shavings measurements showed consistency with the homogeneity requirement of better than 2 % for ^60^Co and than 5 % for ^110m^Ag.

## INTERLABORATORY COMPARISON TO DETERMINE ACTIVITY

The reference activity in the tubes metal standard was determined by means of an interlaboratory comparison on measurement of ^110m^Ag and ^60^Co activity concentration in the 10 disc samples. The comparison was coordinated by JRC, and participants were NCBJ (three discs), PTB (three discs) and JRC-IRMM (four discs).

The calibration procedure of the three laboratories is described in Table 2. Two of the three laboratories derived an experimental efficiency curve using a standard source and calculated efficiency transfer (ET) factors to correct the efficiencies of the reference source for the disc sample geometry. They both derived the ET factors employing validated detector models using different Monte Carlo codes {EGSnrc^(3)^ and Gespecor [http://www.gespecor.de/en/ (12 March 2015, date last accessed)]}. The third laboratory applied a pure experimental calibration using a reference standard which matched the geometry and matrix of the disc sample. In all laboratories, true coincidence summing corrections for both nuclides in question were calculated using the developed detector models as described in Table 2.

**Table 2.** Overview of calibration procedures used.

Procedure	JRC-IRMM	PTB	POLATOM
Setting up experimental efficiency curve	Liquid solution from NPL with five radionuclides	Liquid solution of a PTB mixed standard source with eight radionuclides	Multi-nuclide source of steel discs with spiked foils in between
Setting up computer model	Measuring point sources from PTB and adjusting model parameters	Measuring point sources from PTB and adjusting model parameters	Dimensions defined by Canberra as part of their ISOCS calibration
ET	Detector model and MC simulation with EGSnrc v4-r2.4.0 and general code ‘hpge’^(4, 5)^	Detector model and MC simulation with GESPECOR v4.2	NA
Corrections for TCS	Detector model and calculation of peak efficiency ratios between TCS and NCS MC simulations	Detector model and calculation with GESPECOR v4.2	Detector model and Canberra software ISOCS

TCS, true coincidence summing—simulation of the decay scheme of ^110m^Ag and ^60^Co for the source particles; NCS, no coincidence summing—simulation of a single gamma-emitting source.

**Figure 4.**
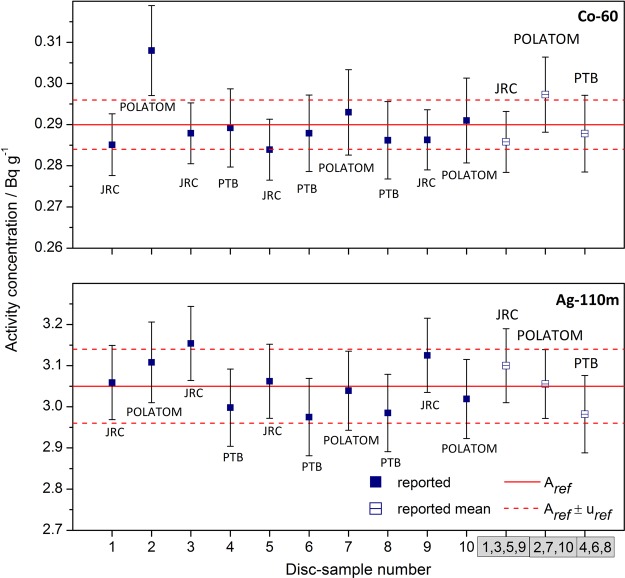
Activity concentrations on 30 September 2013 for ^60^Co and ^110m^Ag in the 10 discs as reported by the three laboratories: JRC, POLATOM and PTB. Solid data points represent results for individual discs whereas empty data points represent the reported mean by each laboratory. The solid lines represent the reference values, *A*_ref_, and the dashed ones their combined standard uncertainties, u_ref_.

The results of the measurements are presented in Figure 4. The combined standard uncertainty^(6)^ (*k* = 1) for the activity concentration, u_char_, was calculated and is shown in Figure 4. The reported JRC result for each of the four discs is the mean value of the measurements on two detectors.

The reference activity concentrations of ^60^Co and ^110m^Ag in the metal standard were calculated using the method of the Power-moderated mean^(7)^ from the mean activity concentrations reported for each nuclide by the three laboratories over their analysed samples. This algorithm considered none of the numbers as outlier and gave the reference values of 0.290±0.006 Bq g^−1^ for ^60^Co and 3.05±0.09 Bq g^−1^ for ^110m^Ag (reference date: 30 September 2013). In the estimation of the combined standard uncertainty of the reference value, u_ref_, the standard uncertainty contribution from the homogeneity study, u_hom_ (see Table 1), was taken into account, along with u_char_, using the following formula:

(1) $${\text{u}}_{\mathrm{ref}}=\sqrt{{\text{u}}_{\mathrm{char}}^{2}+{\text{u}}_{\mathrm{hom}}^{2}}.$$

Uncertainty due to instability is disregarded on the basis of the type of the material that does not allow changes with time and transport.

The final reference values for ^60^Co and ^110m^Ag are given in Table 3 along with their relative combined standard uncertainties. From the reference activity concentrations, the total activity, at the reference date of 30 September 2013, of the 12 tubes, consisting the metal waste reference standard was 71.3±1.4 kBq for ^60^Co and 749±22 kBq for ^110m^Ag. For the mixture of the two radionuclides, the weighted sum of nuclide-specific activity concentrations divided by the corresponding exemption value, of 10 Bq g^−1^ for each of ^60^Co and ^110m^Ag in moderate amounts of material^(1)^, is less than unity and, therefore, the metal standard can be exempted from regulatory control according to the relevant EU Council Directive^(1)^. The standard was measured for 3600 s using the prototype FRMF and resulted in count rates of 120 imp s^−1^ at 658 keV and 15 imp s^−1^ at 1174 keV.

**Table 3.** Reference activity values for ^60^Co and ^110m^Ag on 30 September 2013.

Radionuclide	Activity concentration Bq g^-1^	u_char_ %	u_hom_^a^ %	u_ref_ %	Total activity of the 12 tubes kBq
^60^Co	0.290±0.006	1.69	1.06	1.99	71.3±1.4
^110m^Ag	3.05±0.09	1.69	2.39	2.92	749±22

^a^See Table 1.

## SUMMARY AND DISCUSSION

A new large-volume metal reference standard has been developed for calibration of free-release radioactivity measurement systems. The standard has the form of cast iron tubes, containing certified activity concentrations of ^60^Co and ^110m^Ag, and is placed inside a Euro-pallet sized box. During the standard's design stage, ^60^Co was expected to be distributed homogeneously in the melted iron. Nevertheless, for ^110m^Ag, there was a concern, rising from the less-documented experience with this isotope, that it would not mix so well in the standard's matrix which in turn might give rise to an inhomogeneous activity distribution. From the results of ^60^Co measurements in the discs, presented in Figure 2, one, similar to what was expected, cannot discern any systematic distribution different from the hypothesis of a homogeneous one. However, from the respective results for ^110m^Ag in the same figure, a larger spread is observed originating from the apparent lower, by ∼3 %, ^110m^Ag concentration measured in the discs from the sixth basin as compared with the rest. The results of the shavings' measurements, presented in Figure 3, confirm the above-mentioned observations regarding the superior homogeneity in the distribution of ^60^Co as compared with the one of ^110m^Ag. The higher uncertainty of shavings, which is associated with the sample parameters, as compared with discs, is reflected to the larger spread of data in Figure 3 for both isotopes. Nevertheless, any observed differences in activity concentration are still smaller than what was asked in the criteria for the material. One could possibly expect a difference between the inside and the outside of a tube, but this was not studied as the overall effect of such an inhomogeneity in the standard, consisting of all the 12 tubes, on detection efficiency would be negligible. It would, however, qualify for a future study to determine any inhomogeneities in radial direction arising from centrifugal casting.

When comparing Figure 2 and Figure 3, it is noteworthy that the ^110m^Ag activity distribution seems to show a similar pattern. It indicates that two tubes have slightly lower ^110m^Ag activity concentration than the other tubes, however still within the set requirements of better than 2 and 5 % for ^60^Co and ^110m^Ag, respectively. The adopted inhomogeneity indexes were taken into account in the estimation of the uncertainties of the reference values.

A major drawback of the ^110m^Ag is of course the relatively short half-life of 250 days. The ^110m^Ag activity on the first day (30 July 2013) the standard reached the FRMF was 890 kBq. However, the standard should be possible to use for several years albeit longer measurement times may be required at later stages. It is of course possible to increase the initial activity of ^110m^Ag but that poses additional concerns with respect to radiological safety during production and handling.

Furthermore, a few tests were made by placing these tubes inside waste drums. In a 200-l waste drum, which is typical for waste characterisation facilities, eight of the tubes can nicely be positioned. This gives the user some flexibility of calibrating both waste drums and Euro-pallet-sized waste streams.

## FUNDING

This work was supported by the ENV09 MetroRWM project under the European Metrology Research Programme (EMRP). The EMRP is jointly funded by the EMRP participating countries within EURAMET and the European Union.
